# Automated Analysis of Crackles in Patients with Interstitial Pulmonary Fibrosis

**DOI:** 10.1155/2011/590506

**Published:** 2010-12-21

**Authors:** B. Flietstra, N. Markuzon, A. Vyshedskiy, R. Murphy

**Affiliations:** The Charles Stark Draper Laboratories, Massachusetts Institute of Technology, Faulkner Hospital, 1153 Centre Street, Suite 4990 Boston, MA 02130, USA

## Abstract

*Background*. The crackles in patients with interstitial pulmonary fibrosis (IPF) can be difficult to distinguish from those heard in patients with congestive heart failure (CHF) and pneumonia (PN). Misinterpretation of these crackles can lead to inappropriate therapy. The purpose of this study was to determine whether the crackles in patients with IPF differ from those in patients with CHF and PN. *Methods*. We studied 39 patients with IPF, 95 with CHF and 123 with PN using a 16-channel lung sound analyzer. Crackle features were analyzed using machine learning methods including neural networks and support vector machines. *Results*. The IPF crackles had distinctive features that allowed them to be separated from those in patients with PN with a sensitivity of 0.82, a specificity of 0.88 and an accuracy of 0.86. They were separated from those of CHF patients with a sensitivity of 0.77, a specificity of 0.85 and an accuracy of 0.82. *Conclusion*. Distinctive features are present in the crackles of IPF that help separate them from the crackles of CHF and PN. Computer analysis of crackles at the bedside has the potential of aiding clinicians in diagnosing IPF more easily and thus helping to avoid medication errors.

## 1. Introduction

Crackles are a common finding in patients with interstitial pulmonary fibrosis (IPF). Their presence in a patient is often the first clue that the disease is present. Unfortunately, they can be misinterpreted as being due to congestive heart failure (CHF) or pneumonia (PN), and as a consequence patients may receive inappropriate therapy. On occasion, this can lead to serious, unwanted side effects such as dehydration due to the inappropriate administration of diuretics or an adverse reaction to an antibiotic that was not indicated in the first place. In an attempt to reduce these complications, we studied the sound patterns of patients with these diseases using a multichannel lung sound analyzer (STG16) to determine if such analysis could help differentiate IPF from CHF and PN.

Using advanced statistical techniques we compared features of IPF crackles to those in patients with CHF and PN. Our goal was to determine if there are features of the lung sounds in IPF patients that would help to distinguish them from the lung sounds of patients with CHF and PN.

## 2. Materials and Methods

Patients were selected for this study from a pool of patients who had undergone lung sound analysis as a part of a broader study of the correlation of disease processes with lung sounds patterns. To acquire patients into this study, we identified hospitalized patients and outpatients of a community teaching hospital who were diagnosed as having a specific cardiopulmonary disease or were considered to be normal by their caregivers. The studies were not made on consecutive patients; this is a convenience sample and we currently have over 1,000 patients for whom we have both the diagnosis and the lung sound analysis. The diagnostic category of each of the patients was that of the clinicians caring for these patients. The CHF and PN patients were inpatients in a teaching hospital, and diagnoses were confirmed by board certified specialists. The IPF patients were outpatients and were all seen by pulmonary specialists. There were 39 patients with IPF, 95 with CHF, and 123 with PN. All patients were examined using a multichannel lung sound analyzer (STG16). The details of this device have been described [1]. In brief, patients are asked to lie on a soft foam pad, which has stethoscope chest pieces embedded in it. Each of these chest pieces contains a microphone. The sounds detected by these microphones are amplified, filtered, and input into a computer for analysis. In our usual practice, patients are asked to perform several breathing maneuvers: normal breathing, deeper than normal breathing, coughing, and a vital capacity maneuver. In this study, we chose the data obtained during the deeper than normal breathing maneuver. 

Crackles were defined in accordance with accepted criteria [2, 3]. The STG software automatically identified crackles in all full breaths. The validation of the use of the device as a crackle counter has been reported [4]. A single recording lasted 20 seconds and typically contains a minimum of 3 breaths. To develop algorithms for testing, the crackle features shown in Table 1 were assessed.

**Figure 1:**
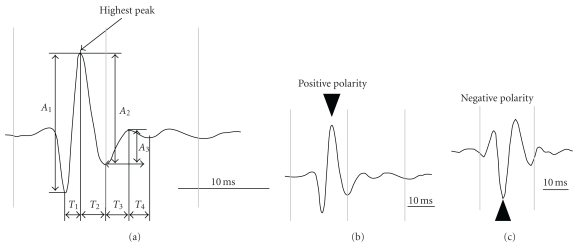
The waveform of a typical crackle (a). The crackle analysis starts by identification of the crackle's highest deflection highest peak. The half-period to the left of the highest peak is marked as *T*_1_. The half-period to the right of the highest peak is marked as *T*_2_. Crackle pitch is calculated from 4 consecutive half-periods, with *T*_1_ as a 1st half-period. The amplitude is determined separately for each half-period and marked as *A*_1_, *A*_2_, and *A*_3_. Crackle polarity (b) crackle polarity is defined positive if the highest peak is upward (c). Crackle polarity is defined negative if the highest peak is downward.

Crackle features were calculated separately for inspiratory crackles and for expiratory crackles.  Figure 1 demonstrates the process of calculating features of the crackle. In addition to these features, we combined the individual crackle features in the form of a median (median T1, median pitch, etc.)

In addition to features based on individual crackle properties we captured information reflecting the distribution of the patient's crackles. Diseases differ in the pattern of crackles distribution over the chest. Distribution information required aggregation of data on a per-breath level and led to the development of aggregate crackle features shown in Table 2.

To perform classification and prediction we utilized supervised learning nonparametric classifiers: neural networks and support vector machines [5, 6]. Supervised learning can teach the system to nonlinearly map the input features to the associated label of disease. We divided the data into a training set, used for feature extraction and model building, and a validation set, used for evaluation of the results. Validation data set performance indicates how well the features generalize to the unseen data. We used a fivefold cross-validation to increase the pool of validated data.

We used individual crackle features to distinguish crackles of IPF from CHF crackles and PN crackles. Once individual crackles were classified as IPF, CHF, or PN, majority voting was used to classify the patient into one of the three disorders. To incorporate features of crackle distribution, we performed majority voting among individual breaths during the single recording; for example, if a patient had 6 breaths, and 3 of them were classified as IPF, 2 as CHF, and 1 as PN, then the patient would be classified as having IPF. The final classification of IPF versus CHF and IPF versus PN was performed using this breath majority voting.

The study was approved by the Institutional Review Board of the Brigham and Women's/Faulkner Hospitals. 

## 3. Results

**Figure 2:**
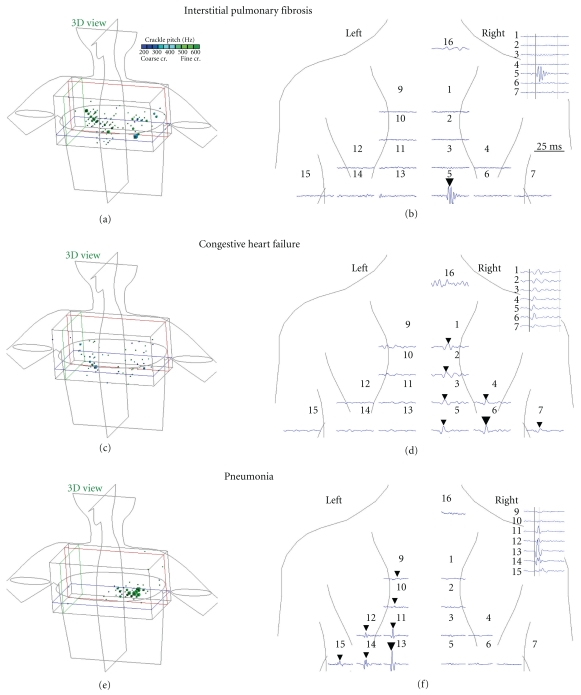
Examples of lung sound analysis in three individual patients. Left panel: based on arrival time differences at the microphones all crackles were localized inside the chest. Crackles are displayed as cubes overlaid on the three-dimensional display. The size of each cube is proportional to the crackle density. Crackle pitch is color coded: the insert shows the legend. Right panel: to illustrate the difference in crackle transmission, an individual crackle is shown in the right panel. Sound waveforms are shown as detected in the microphones arrayed over the posterior chest. The IPF crackle is only detected by one microphone, while the CHF and PN crackles are detected by several microphones. The insert shows the crackle waveforms in stacked mode to facilitate examination of arrival times at the various microphones.

Figure 2 shows crackle analysis in the three representative patients with IPF, CHF, and PN. The left panels show three-dimensional models of the thorax with crackles overlaid on the three-dimensional display. Crackles are displayed as cubes. The size of each cube is proportional to the crackle density. The patient with IPF had over 100 crackles recorded over 20 seconds, panel (a). The crackles localized in three-dimensional space are distributed uniformly. The patient with CHF had over 50 crackles distributed with accentuation toward lung bases, panel (c). The patient with PN had over 70 crackles localized to the left lower lobe where radiography revealed opacifications, panel (e).

The display of a single crackling event reveals that the crackling sound is transmitted differently in the three diseases. The right panels in Figure 2 show time-expanded sound waveforms that were recorded by the 14 microphones positioned over the posterior chest. The waveforms are superimposed on a body plot. Each waveform is positioned on the part of the body where the sound was recorded. 

In the patient with CHF, panel (d), a prominent crackle is seen on the tracing from channel 6 (indicated by a large triangle). At the same time the crackling sound was also detected in all ipsilateral microphones 1, 2, 3, 4, 5, and 7 (marked by triangles). The set of crackles generated by a single event and recorded by multiple microphones is referred to as a crackle family [7, 8]. The crackle waveforms corresponding to the crackle family are shown in the stack mode in the insert in the upper-right corner. Notice that the crackle recorded by microphone 6 (the most prominent crackle or mother crackle) occurs earlier than the other crackles. 

The crackle transmission coefficient was calculated for each crackle family. In the crackle family shown in the CHF patient (Figure  2(d)), the CTC was 50%. (The CTC has a value of 0% in the absence of any transmission and 100% when there is equal transmission to all ipsilateral channels.) In the crackle family shown in the PN patient, Figure  2(f), the CTC was 16%. In contrast, the crackle in the IPF patient was detected at only a single microphone (Figure  2(b)). The CTC of this crackle family was 1%. The low CTC is typical in IPF patients.

In addition to the CTC, note the difference in the pitch of the crackles shown in Figure 2, right panels: 588 Hz in IPF versus 218 Hz in CHF and 364 Hz in PN. Also note the difference in the number of zero crossings: 15 in IPF versus 5 in CHF and 5 in PN. 

The observations in single patients shown in Figure 2 are supported by statistical analysis of all available data.  Table 3 shows crackle rate and individual crackle features in IPF, CHF, and PN. Note that multiple individual crackle features are significantly different between IPF and the other two diseases.

In order to perform classification of patients into one of the three diseases we utilized two statistical methods: neural networks and support vector machines.  Table 4 presents the results of binary comparisons of individual crackles in IPF versus CHF and IPF versus PN. As seen in Table 4, the sensitivity, specificity, and overall accuracy are over 70%, consistent with the conclusion that individual IPF crackles have features that differ from those of patients with PN and CHF. 

The accuracy increased to 83% (Table 5) on the application of majority voting to the classification of individual breaths based on crackle features. The addition of aggregate crackle features improved the accuracy to 86% (Table 6). Finally, we used majority voting to classify patients based on crackle features (Table 7). The performance of per-breath and that of per-patient classification are quite similar suggesting that most breaths of the same patients are classified in a similar manner. 

## 4. Discussion

This study shows that the crackles of IPF have features that help distinguish them from the crackles of patients with CHF and PN. As noted, we believe that the crackles of IPF are not infrequently misinterpreted. They are most commonly considered to be due to CHF, and diuretics are administered inappropriately. There is not much literature to support this observation, but it is our personal experience and an informal survey of clinicians confirmed this opinion. In addition to providing evidence that helps in accurately identifying IPF crackles, computerized lung sounds analysis also quantifies them. It has long been noted that crackles of IPF become more widespread when the disease progresses. Thus crackle quantification can be important in assessing the severity of IPF, and this could be useful in providing evidence of response to therapy. 

We focused on the difference between crackles of IPF and those of CHF and PN. Baughman et al. took a different approach. They showed that the presence of crackles could help clinicians in distinguishing sarcoidosis from IPF [9]. Crackles were much less numerous in patients with sarcoidosis than in those with roentgenologically equivalent severity of changes due to IPF.

Among features that are significantly different between IPF and CHF/PN is the crackle pitch (*P* < .0000001). This is consistent with the commonly held believe that the crackles of IPF are generated in smaller airways than those of CHF and PN. The distinctive features of crackles of IPF have been long recognized. For example, the crackles of pulmonary fibrosis caused by asbestos, described in early as 1930 by Wood and Gloyne to be a prominent feature of this industrial disease, were described by Smither as “characteristic in their sound and distribution” [10, 11]. He also pointed out that they are present first at the bases in the midaxillary line and then tend to spread to the posterior bases. As the disease progresses, crackles become audible higher on the chest. In one study a technician was able to screen workers for asbestosis by detecting crackles. The technician correctly identified all workers in whom the diagnosis was most certain, that is, those with all the clinical, physiological, and roentgenologic criteria used in the study [12]. Using time-expanded waveform analysis, Kawamura et al. studied 18 patients with IPF and 23 patients with crackles who did not have this disease. Two crackle parameters (initial deflection width and two cycle duration) were shorter in the IPF patients. This finding correlated with HRCT findings in these patients [13]. British investigators have reported that detecting crackles on time-expanded waveform analysis was equivalent to CT scans in detecting asbestosis [14]. Finnish investigators also showed a significant positive correlation with frequencies of lung sounds and pulmonary fibrosis detected on HRCT [15]. Of course in industrial settings, in contrast to ER's and ICU's, neither CHF nor PN crackles are likely to be confounding variables.

To perform classification and prediction, we utilized well-established supervised learning nonparametric classifiers: neural networks and support vector machines [5, 6]. Neural networks (NNs) are the name for non-linear statistical data modeling tools. They are used to model complex relationships between inputs and outputs and are an attempt to build an architecture similar to the one of the human brain. NNs consist of an interconnected group of artificial neurons that learns and updates its internal structure using a connectionist approach to computation. NNs utilize a data-driven approach where changes in internal structure are based on external or internal information that flows through the network during the learning phase. In this study, we used a back propagation neural network. Support Vector Machines (SVM) are one of the newest methods in the supervised learning field. Generally speaking, a support vector machine seeks to create a hyperplane in a high-dimensional space that separates the two data classes. Not only does the hyperplane separate the data, but also it is oriented in such a fashion that creates the maximum “margin” on both sides of it ensuring the largest possible separation between the two classes. The algorithm proved to be fast and very efficient. We note here that both NN and SVM classification achieved similar results.

The technology for this study came about in part because there has been resurgence in interest in lung sounds. This has been stimulated by the development of computerized techniques. A number of investigations demonstrating the usefulness of computerized lung sound analysis have been reported [16–21]. While crackle pitch can be assessed by a clinician using an acoustic stethoscope, other crackle features that are significantly different between IPF and CHF/PN can only be gained with the use of a computerized stethoscope. And some crackle features such as crackle transmission coefficient can only be calculated with the use of a multichannel lung sound analyzer.

Computerized lung sound analysis can now be done at the bedside. The examinations are easy to do and can be performed in a few minutes. They have been shown to help in the detection of pneumonia [22]. Unfortunately, devices capable of doing this are not currently widely available. However, it is likely that this will change as the advantages of this technology become more widely known. Used in the context of a complete medical evaluation, we believe that this information could help avoid misinterpretation of IPF crackles and thus potentially decrease the occurrence of inappropriate treatment. 

## Figures and Tables

**Table 1:** 

Individual crackle features	Definition
Number of zero line crossings (ZXS)	The number of times the crackle waveform crossed the baseline
*T*_1_	First half-period, Figure 1(a)
Crackle pitch	Crackle pitch (spectral frequency) calculated from 4 half-periods: *T*_1_, *T*_2_, *T*_3_, and *T*_4_, Figure 1(a)
*T*_2_/*T*_1_	Ratio of the 2nd and 1st half periods
Half-period duration variability (%)	(Standard deviation {*T*_1_, *T*_2_, *T*_3_,…, *T*_*n*_} × 100%)/(mean {*T*_1_, *T*_2_, *T*_3_,…, *T*_*n*_})
Crackle timing (timing)	Crackle timing is defined as follows: 1 for early inspiration, 2 for mid-inspiration, 3 for late inspiration, 4 for early expiration, 5 for mid-expiration, 6 for late expiration
Crackle transmission coefficient (CTC)	The degree of crackling sound transmission through the ipsilateral chest, as calculated from crackle family observation by multiple microphones. The CTC has a value of 0% in the absence of any transmission and 100% when there is equal transmission to all ipsilateral channels see [7] for detailed description and discussion.
Amplitude	Amplitude of the highest peak (arbitrary units)
*A*_2_/*A*_1_	See Figure 1(a)
*A*_3_/*A*_1_	See Figure 1(a)
Half period amplitude variability (%)	(Standard deviation {*A*_1_, *A*_2_, *A*_3_,…, *A*_*n*_} × 100%)/(Mean {*A*_1_, *A*_2_, *A*_3_,…, *A*_*n*_})
Crackle polarity (polarity)	Direction of the highest peak, Figures 1(b) and 1(c) see [8] for detailed description and discussion

**Table 2:** 

Aggregate crackle features	Definition
Number of crackles per breath (Cr/breath)	The total number of crackles per breath as detected by the computer
Number of crackles per breath per quadrant (top left, top right, bottom left, bottom right)	These 4 features count the total number of crackles observed in each quadrant of the chest. Together they add up to the total number of crackles per breath
Percentage differences between crackle quadrants (6 total)	Calculated from the 4 features described above, these features represent a comparison between quadrants. Each percentage is a pairwise comparison of all 6 possible combinations of quadrants
Maximum distances (*x*,*y*,*z*)	Distances between crackles in 3-dimensional space. There are separate features for *x*, *y*, and *z* planes. One feature also records a maximum distance across all 3 dimensions
Channel distances	These features are similar to those described above, except that they are defined based upon which channel microphone picked up the crackle. Distances are defined accordingly

**Table 3:** Crackle rate and individual crackle features in IPF, CHF, and PN. The results are presented as means ± SD. Student's *t*-test was used to compare the variables between the groups. Values of *P* that are less than.05 are shown in bold.

	Crackle Features	IPF (*n* = 39)	CHF (*n* = 95)	PN (*n* = 123)	IPF versus CHF	IPF versus PN
Inspiration	Crackle rate (crackles per breath)	18 ± 14	7 ± 5	7 ± 4	*P* < .0001	*P* < .0001
	Crackle pitch (Hz)	416 ± 88	302 ± 64	284 ± 60	*P* < .0000001	*P* < .0000001
	*T*_1_ (s)	1.2 ± 0.2	1.4 ± 0.2	1.5 ± 0.3	*P* < .0000001	*P* < .0000001
	Number of zero line crossings (ZXS)	9 ± 2	6 ± 1	6 ± 1	*P* < .0000001	*P* < .0000001
	*T*_2_/*T*_1_	1.2 ± 0.1	1.5 ± 0.3	1.5 ± 0.2	*P* < .00001	*P* < .00001
	Half-period duration variability (%)	38 ± 8	37 ± 9	37 ± 7	*P* = .57	*P* = .57
	Crackle timing (Timing)	2.1 ± 0.3	2.1 ± 0.3	2.0 ± 0.4	*P* = .98	*P* = .29
	Crackle transmission coefficient (CTC)	16 ± 5	23 ± 6	23 ± 7	*P* < .0000001	*P* < .0000001
	Amplitude	9 ± 9	6 ± 5	7 ± 7	*P* = .06	*P* = .34
	*A*_2_/*A*_1_	1 ± 0.1	1 ± 0.1	1 ± 0.1	*P* = .24	*P* = .02
	*A*_3_/*A*_1_	0.5 ± 0.1	0.4 ± 0.1	0.4 ± 0.1	*P* < .0000001	*P* < .0000001
	Half-period amplitude variability (%)	68 ± 11	53 ± 12	48 ± 12	*P* < .0000001	*P* < .0000001
	Crackles with positive polarity (%)	74 ± 13	67 ± 20	70 ± 19	*P* = .02	*P* = .18

Expiration	Crackle rate (crackles per breath)	9 ± 7	5 ± 3	5 ± 5	*P* = .01	*P* = .07
	Crackle pitch (Hz)	411 ± 71	289 ± 65	264 ± 77	*P* < .000001	*P* < .000001
	*T*_1_ (s)	1.2 ± 0.3	1.6 ± 0.3	1.8 ± 0.3	*P* < .000001	*P* < .000001
	Number of zero line crossings (ZXS)	10 ± 2	7 ± 1	7 ± 2	*P* < .000001	*P* < .000001
	*T*_2_/*T*_1_	1.3 ± 0.1	1.4 ± 0.2	1.4 ± 0.3	*P* = .001	*P* = .002
	Half-period duration variability (%)	43 ± 14	39 ± 8	37 ± 9	*P* = .17	*P* = .05
	Crackle timing (Timing)	5.2 ± 0.3	5.1 ± 0.4	5.0 ± 0.3	*P* = .25	*P* = .13
	Crackle transmission coefficient (CTC)	18 ± 7	25 ± 9	27 ± 9	*P* < .001	*P* < .00001
	Amplitude	5 ± 7	5 ± 4	6 ± 5	*P* = .72	*P* = .44
	*A*_2_/*A*_1_	1.0 ± 0.1	1.1 ± 0.1	1.1 ± 0.1	*P* = .11	*P* = .01
	*A*_3_/*A*_1_	0.5 ± 0.1	0.4 ± 0.1	0.4 ± 0.2	*P* = .006	*P* = .002
	Half-period amplitude variability (%)	70 ± 14	49 ± 14	46 ± 13	*P* < .00001	*P* < .0000001
	Crackles with positive polarity (%)	34 ± 17	44 ± 25	33 ± 24	*P* = .10	*P* = .80

**Table 4:** Crackle classification for IPF versus PN and IPF versus CHF using individual crackle features.

	SVM			Neural networks		
	Sensitivity	Specificity	Accuracy	Sensitivity	Specificity	Accuracy
PN	0.80	0.71	0.74	0.74	0.78	0.75
CHF	0.79	0.73	0.78	0.78	0.77	0.78

**Table 5:** Breath classification for IPF versus PN and IPF versus CHF using individual crackle features. The model was created using NNs and voting over classifications of individual crackles in a breath.

	Crackle only		
	Sensitivity	Specificity	Accuracy
PN	0.76	0.84	0.83
CHF	0.78	0.84	0.83

**Table 6:** Breath classification for IPF versus PN and IPF versus CHF using individual and aggregate crackle features. The model was created using NNs and voting over classifications of individual crackles in a breath.

	Crackle and distribution		
	Sensitivity	Specificity	Accuracy
PN	0.76	0.87	0.86
CHF	0.78	0.89	0.88

**Table 7:** Patient classification for IPF versus PN and IPF versus CHF using individual and aggregate crackle features and majority voting.

	SVM voting			Neural networks voting		
	Sensitivity	Specificity	Accuracy	Sensitivity	Specificity	Accuracy
PN	0.82	0.88	0.86	0.77	0.91	0.88
CHF	0.77	0.85	0.82	0.78	0.88	0.85
